# Integrative taxonomy of *Metastrongylus* spp. in wild boars from Brazil

**DOI:** 10.1186/s13071-023-06047-x

**Published:** 2023-12-05

**Authors:** Wilson Junior Oliveira, Patricia Parreira Perin, Carmen Andrea Arias Pacheco, Talita Oliveira Mendonça, Andressa de Souza Pollo, Renan Bressianini do Amaral, Natália de Oliveira Zolla, Lívia de Oliveira Andrade, Jonathan Silvestre Gomes, Vitória Maximiana Soares dos Santos, Adrian Felipe de Moraes Ferreira, Estevam Guilherme Lux Hoppe

**Affiliations:** 1 São Paulo State University, Jaboticabal, São Paulo Brazil; 2 University of São Paulo, Ribeirão Preto, São Paulo Brazil

**Keywords:** Invasive species, Morphology, Neotropical region, Lungworms, Phylogeny, *Metastrongylus*

## Abstract

**Background:**

Wild boars *(Sus scrofa)* may cause substantial damage to crops and can spread zoonotic parasites to domestic animals, posing a risk to health and animal production. *Metastrongylus* spp. can negatively affect the wild boar population, increasing piglet mortality. In addition to that, studies with *Metastrongylus* genetic characterization are still scarce in Brazil. The present study aims to characterize *Metastrongylus* spp. from wild boars hunted in the states of São Paulo, Paraná, and Rio Grande do Sul, Brazil, using traditional morphological description and DNA sequences in an integrative taxonomic approach.

**Methods:**

After nematode collection from 58 wild boars, the parasites were morphologically identified and genetically characterized by the amplification of 18S ribosomal DNA (rDNA), 28S rDNA, internal transcribed spacer (ITS) region, and *cox-1* mitochondrial DNA (mtDNA). Descriptors of infection were determined and Pearson's Chi-square test was applied to compare the prevalence of infections among the identified parasite species, host age group (juveniles and adults), and sex. The Mann–Whitney *U* test was performed to compare the mean intensity between the age groups and sex.

**Results:**

*Metastrongylus salmi*, *Metastrongylus apri*, and *Metastrongylus pudendotectus* were identified in 77.6% (45/58) of the necropsied wild boars. *Metastrongylus salmi* was the most prevalent and abundant species (70.7%, 11.1), followed by *M. pudendotectus* (18.9%, 4.3) and *M. apri* (17.2%, 2.2). *Metastrongylus pudendotectus* showed the highest mean intensity and range (25.2, 1–93), followed by *M. salmi* (15.7, 1–58) and *M. apri* (12.6, 3–27). We found a significantly higher prevalence of *Metastrongylus* spp. and *M. salmi* in adult wild boars, probably associated with a more prolonged time of exposure to intermediate host species. The phylogenetic analysis revealed that ITS2 region and *cox-1* mtDNA are the most suitable genetic markers for *Metastrongylus* species characterization. Genetic variability between *M. apri* and *M. salmi* isolates was verified.

**Conclusions:**

We expand the knowledge about the *Metastrongylus* community in the non-captive wild boar population from Brazil as well as the importance of this exotic species in the maintenance of *Metastrongylus* spp. in its areas of occurrence. The novel genetic sequences obtained may help further studies to understand the genetic diversity in other nematode populations from Brazil and other countries.

**Graphical Abstract:**

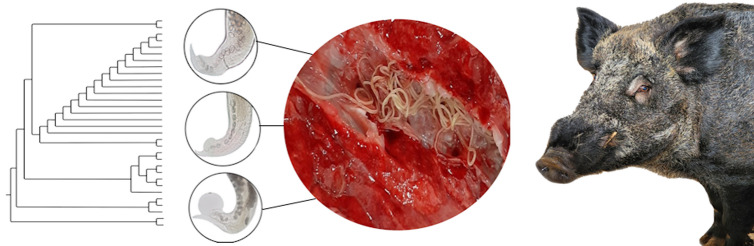

**Supplementary Information:**

The online version contains supplementary material available at 10.1186/s13071-023-06047-x.

## Background

Wild boars (*Sus scrofa*) are invasive species widely distributed in Brazil. These animals are related to negative impacts on natural and agricultural environments, in addition to spreading zoonotic parasites [1, 2] posing a risk to the health of domestic animals and the conservation of native species [3]. Furthermore, wild boar populations are negatively affected by *Metastrongylus* spp. lungworms, increasing piglet mortality in particular [4, 5].

Like other correlated species, *Metastrongylus* have a heteroxenic life cycle with several earthworm species acting as intermediate hosts [6]. The parasite eggs ingested by earthworms develop into third-stage larvae in their tissues. After wild boars ingest the intermediate host, the parasite migrates through the mesenteric lymph nodes and the right heart to the lungs, reaching the adult form in the lumen of the bronchi and bronchioles [7]. The genus comprises six species: *Metastrongylus salmi*, *M. apri* (syn. *M. elongatus*), *M. pudendotectus*, *M. confusus*, *M. asymmetricus*, and *M. madagascariensis*, which may occur in mixed infections. All species are described in wild and domestic suids except *M. madagascariensis*, which is only found in domestic pigs from Madagascar [7]. In Brazil, *M. salmi*, *M. pudendotectus*, and *M. apri* are reported [8].

Currently, *Metastrongylus* spp. genetic sequences available in databases such as GenBank are still limited, compromising studies that aim to describe the genetic variability or make phylogenetic inferences. Studies focusing on helminth genetic variability are scarce worldwide and almost nonexistent in Brazil [8]. Thus, the present study aims to characterize *Metastrongylus* spp. from wild boars from the states of São Paulo, Paraná, and Rio Grande do Sul, Brazil, using traditional morphological description and DNA sequences in an integrative taxonomic approach.

## Methods

### Study areas

**Fig. 1:**
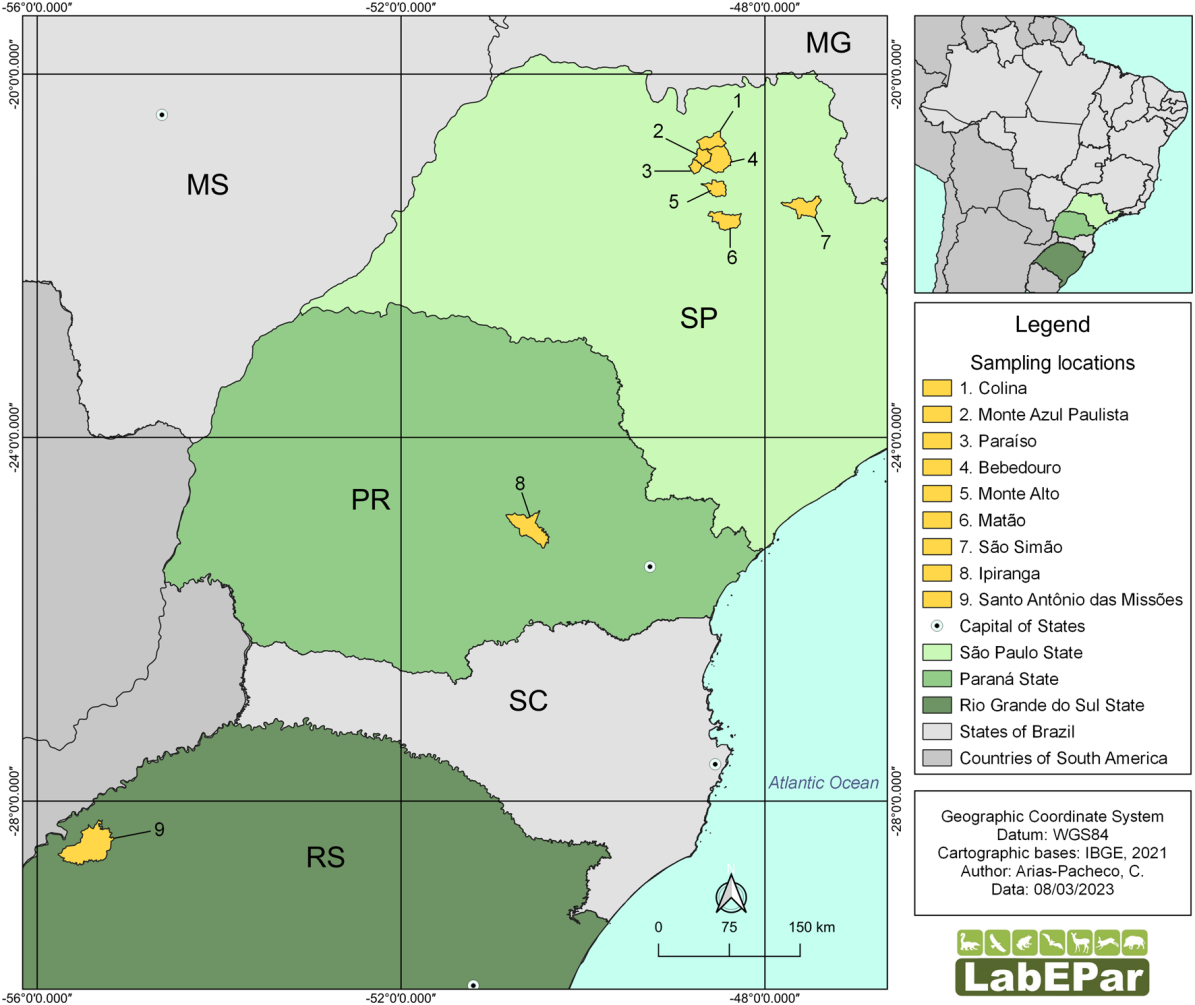
Sampling collection sites of wild boars hunted in São Paulo, Paraná, and Rio Grande do Sul states

The samples were collected from wild boars hunted in rural properties from the municipalities of São Simão, Monte Azul, Paraíso, Colina, Matão, Bebedouro e Monte Alto (São Paulo), Ipiranga (Paraná), and Santo Antônio das Missões (Rio Grande do Sul) (Fig. 1).

The study areas in São Paulo state are located in the transition zone between the Cerrado and the Atlantic Forest biomes. According to the modified Köppen climate classification, the climate is humid subtropical, with an average annual temperature above 18 °C and average annual rainfall between 1200 and 1500 mm. They have 4–5 months of drought in winter, between May and September, and are located about 600 m above sea level [9–11]. Agriculture is the main economic activity, with emphasis on sugarcane, soy, corn, peanut, and tomato crops, in addition to beef cattle and poultry [12].

In Paraná State, the Campos Gerais region, where Ipiranga is located, has an average annual rainfall between 1400 and 1800 mm and an average annual temperature between 16 ºC and 20 ºC. The wettest season is from September to March, but frequent precipitation occurs during the winter. The prevailing climate is humid subtropical, according to the Köppen classification, and is located at about 800 m above sea level. The characteristic vegetation is the mixed rainforest. The region is characterized by high-tech crops such as soy, corn, wheat, potatoes, and beans, in addition to dairy cattle [13].

The municipality of Santo Antônio das Missões belongs to the Missões region, northwest of Rio Grande do Sul state, located in the Pampa biome. According to the Köppen classification, the climate is humid subtropical with an average annual temperature of about 17 ºC. January is the hottest month (average 32.7 ºC), and July is the coldest (average 10.5 ºC). Rainfall is about 1900 mm/year, with uneven distribution during the period. The economy is based on agricultural products, with emphasis on soy, rice, wheat, corn, sheep, and beef and dairy cattle, in addition to diversified subsistence production [14].

### Biological samples

The sampling was carried out without biostatistical criteria due to the lack of data regarding the wild boar population in the region. Instead, it relied on the hunting success of our partner hunters. We examined the lungs of 58 wild boars, comprising 33 males and 25 females. The age of the animals was estimated according to dental eruption [15], categorizing them as either juveniles (less than 6 months old), or adults (more than 6 months old). The classification of the two age groups was established based on our fieldwork observations of pregnant wild boars around 6 months old (EGL Hoppe, personal communication, July 17, 2023) probably due to the random crosses between wild boars and domestic pigs. The organs were removed from the thoracic cavity, packed in individually labeled plastic bags, stored in isothermal boxes with ice, and immediately sent to the Laboratory of Parasitic Diseases (LabEPar) at the Department of Pathology, Reproduction and One Health (DPRSU), within the School of Agricultural and Veterinary Studies (FCAV), at the São Paulo State University (Unesp), Jaboticabal, São Paulo, Brazil.

### Morphological identification

The trachea and the lungs were slit opened following the airways, from the trachea and main bronchi to the terminal bronchioles. All obtained nematodes were fixed in 70% ethanol and stored in identified flasks. The parasites were clarified with 80% acetic acid and mounted on temporary slides for taxonomic identification, according to Vicente et al. [16] and Gassó et al. [6]. Images and measurements (in millimeters, expressed as mean ± standard deviation, lower and upper values in brackets) were obtained with an Olympus BX-51 microscope attached to a Q-Color 3 camera (Olympus, Tokyo, Japan) and processed using Image-Pro Plus 4 image analyzer software (Media Cybernetics, Rockville, MD, USA). Vouchers were deposited in the collection of the Oswaldo Cruz Institute (CHIOC/Fiocruz, Rio de Janeiro, Brazil), and additional specimens were kept in LabEPar's helminthological collection.

### Molecular analysis

#### DNA extraction

Genomic DNA was extracted from at least two male specimens per municipality studied. Selected specimens were individually washed with sterile phosphate-buffered saline (PBS) pH 7.4 solution and transferred to 1.5 µl microtubes containing 50 µl of tissue lysis buffer (ATL) from the DNeasy Blood & Tissue Kit (Qiagen, Hilden, Germany) and macerated with the aid of sterilized plastic rods. Subsequently, glass beads treated with Triton X-100, 130 µl of ATL buffer, and 20 µl of proteinase K were added to the microtubes. The rest of the extraction proceeded according to the manufacturer’s protocol. The analysis of DNA concentration and quality, whose absorbance ratio between the wavelengths of 260 and 280 nm is desirable between 1.8 and 2.0 ng/dl [17], was performed using the NanoDrop One Spectrophotometer (Thermo Fisher Scientific), and the extraction products were stored at −20 °C until amplification by conventional polymerase chain reaction (PCR).

#### Amplification

Four genetic regions were amplified: 18S ribosomal DNA (rDNA), internal transcribed spacer (ITS), 28S rDNA, and the cytochrome *c* oxidase subunit I (*cox-1*) of the mitochondrial DNA (mtDNA). The primers set are expressed in Table 1. The reactions were composed of 1× buffer (KCl 50 mM, TRIS–HCl 200 mM, pH 8,4); 50 mM of MgCl_2_; 10 mM dNTPs; 0.5 U Platinum Taq [*Thermus aquaticus*] DNA Polymerase (Invitrogen, Thermo Fisher Scientific, Waltham, MA, USA); 5 pmol of each forward and reverse primer; 60 ng of genomic DNA and ultrapure water to complete a final volume of 20 µl. Amplifications were performed in a Nexus thermal cycler (Eppendorf, Hamburg, Germany) programmed to perform one cycle at 95 ºC for 3 min, and 35 cycles at 94 ºC for 40 s; each primer's annealing temperature (Table 1) was kept for 30 s, and 72 ºC for 50 s, followed by a final extension cycle at 72 ºC for 10 min.

**Table 1:** Primer sets and annealing temperature used in polymerase chain reactions with their respective amplicon size

Region	Primer	*T* (ºC)	Amplicon	Sequence (5′–3′)	References
18S1	988F 1912R	50	1500 bp	CTCAAAGATTAAGCCATGC TTTACGGTCAGAACTAGGG	Holterman [57]
18S2	1813F 2646R	52	1500 bp	CTGCGTGAGAGGTGAAAT GCTACCTTGTTACGACTTTT	Holterman [57]
28S	D2A D3B	55	650 bp	ACAAGTACCGTGAGGGAAAGTTG TCGGAAGGAACCAGCTACTA	De Ley et al. [58]
ITS	NC1 NC2	54,5	800 bp	ACGTCTGGTTCAGGGTTGTT TTAGTTTCTTTTCCTCCGCT	Gasser et al. [59]
*cox-1*	F1 R2	45	1015 bp	CCTACTATGATTGGTGGTTTTGGTAATTGGTAGCAGCAGTAAAATAAGCACG	Kanzaki and Futai [60]

*T* annealing temperature, *bp* base pairs

To verify the amplification reaction, the PCR products were submitted to electrophoresis in 1% agarose gel, stained with ethidium bromide, and visualized in a Geldoc XR photodocumenter (Bio-Rad^®^). In the case of low DNA yield, the reamplification was performed using the same protocol and primers cited previously. The products were purified with the Wizard^®^ SV Gel and PCR Clean-Up System kit (Promega, Madison, WI, USA) according to the manufacturer's instruction and submitted to PCR sequencing using the BigDye Terminator v3.1 kit (Applied Biosystems, Waltham, MA, USA), according to manufacturer’s instructions. Sequencing was performed by capillary electrophoresis on an ABI 3130 sequencer (Applied Biosystems, Waltham, MA, USA) according to Sanger's method [18].

### Phylogenetic analysis

The electropherograms generated in the sequencing were submitted to the Phred/Phrap/Consed software package [19–21] to verify the quality of the bases and trim the sequences considering bases with Phred quality up to 20 or higher. The qualified sequences were compared to others deposited in the National Center for Biotechnology Information (NCBI) database using BLAST (Basic Local Alignment Search Tool) [22]. The sequences from this study and the selected sequences from NCBI's database were aligned using the ClustalW tool [23] on the software BioEdit v. 7.0.5.3 [24].

Phylogenetic trees were obtained by a maximum likelihood analysis using the W-IQ-Tree software [25]. The best evolutionary model was selected according to the Bayesian information criterion (BIC) using the W-IQ-Tree software [26]. The clade stability was evaluated using 1000 bootstrap replicates. The phylograms were graphically edited and rooted on the TreeGraph 2.15.0–887 beta software [27].

### Data analysis

Infection descriptors of prevalence, mean intensity (range of intensity), and mean abundance were based on Bush et al. [28]. Fisher’s exact test was performed to compare parasite prevalence among sex, age group, and states of collection. The Mann–Whitney *U* test was used to evaluate the differences in mean intensity between the age groups and sex. Differences between the states’ mean intensity were not calculated due to the low parasite burden observed in Paraná state (only one animal was infected). All analyses were performed using the R software version 4.0.4. Values of *p* < 0.05 were considered statistically significant.

### Ethical procedures

All procedures were approved by the Committee on Ethics in the Use of Animals (CEUA) of FCAV/Unesp Jaboticabal protocol no. 3683/20 and Chico Mendes Institute for Biodiversity Conservation (ICMBio), request in the Biodiversity Authorization and Information System (SISBIO) no. 84726–1.

## Results

### Parasite community

Lungworms were observed in 45 out of 58 animals (77.6%). A total of 1,016 parasites were recovered and three species (*M. salmi*, *M. apri*, and *M. pudendotectus*) were identified. Infection descriptors are shown in Table 2. *Metastrongylus salmi* was the most prevalent and abundant species (70.7%, 11.1), followed by *M. pudendotectus* (18.9%, 4.3) and *M. apri* (17.2%, 2.2). *Metastrongylus pudendotectus* showed the highest mean intensity and range (25.2, 1–93), followed by *M. salmi* (15.7, 1–58) and *M. apri* (12.6, 3–27). In São Paulo state, *M. salmi* was the only species identified. In the southern states (Paraná and Rio Grande do Sul), the three helminth species were present in mixed infections by two or three of them. *Metastrongylus salmi* (odds ratio [OR] = 0.1, 95% confidence interval [CI] = 0.02–0.6, *P* = 0.004) and *Metastrongylus* spp. (OR = 0.08, 95% CI = 0.01–0.4, *P* = 0.0005) prevalence was higher in adults than juveniles (Table 3). No significant differences (*p* > 0.05) were observed between the mean intensities relative to sex and age group (Table 4).

**Table 2:** Prevalence, mean intensity, range of intensity, and mean abundance relative to species and collection states of *Metastrongylus* spp. for wild boars hunted in São Paulo, Paraná, and Rio Grande do Sul states, Brazil

Species/states	Prevalence (%)	Mean intensity	Range of intensity	Mean abundance
*Metastrongylus salmi*	70.7	15.7	1–58	11.1
*Metastrongylus apri*	17.2	12.6	3–27	2.2
*Metastrongylus pudendotectus*	18.9	25.2	1–93	4.3
São Paulo	77.5	16.1	1–58	12.1
Paraná	33.3	^a^21.0	NA	7.0
Rio Grande do Sul	86.7	39.1	1–128	33.9

^a^Intensity (just one animal was infected). *NA* not applicable. Total values are displayed

**Table 3:** Fisher’s exact test results for the comparison of *Metastrongylus* spp. prevalence between states, host sex, and age group for wild boars hunted in São Paulo, Paraná, and Rio Grande do Sul states, Brazil

	States					Sex			Age group		Statistical analysis
	SP (*n* = 40) Pr (%)	PR (*n* = 3) Pr (%)	RS (*n* = 15) Pr (%)	Statistical analysis (SP × PR)	Statistical analysis (PR × RS)	M (*n* = 33 Pr (%)	F (*n* = 25) Pr (%)	Statistical analysis	J (*n* = 13) Pr (%)	A (*n* = 45) Pr (%)	
Ms	77.5	0	73.3	OR = 0, 95% CI = 0, *P* = 1	OR = 0, 95% CI = 0, *P* = 1	72.7	72.0	OR = 1.7, 95% CI = 0.4–7.4, P = 1.0	38.5	82.2	OR = 0.1, 95% CI = 0.02–0.6, *P = a0.004
Ma	0	33.3	60.0	OR = 0, 95% CI = 0, *P* = 1	OR = 0.3, 95% CI = 0.005–8.3, *P* = 0.5	24.2	8.0	OR = 3.6, 95% CI = 0.6–38.3, P = 0.2	0	20.0	OR = 0, 95% CI = 0, *P* = 1
Mp	0	33.3	66.6	OR = 0, 95% CI = 0, *P *= 1	OR = 0.3, 95% CI = 0.003–6.4, *P* = 0.5	24.2	12.0	OR = 2.3, 95% CI = 0.4–15.2, P = 0.4	0	22.2	OR = 0, 95% CI = 0, *P* = 1
Total	77.5	33.3	86.7	OR = 6.5, 95% CI = 0.3–417.7, *P* = 0.1	OR = 0.3, 95% CI = 0.003–6.4, *P* = 0.5	81.8	72.0	OR = 1.7, 95%CI = 0.4–7.4, P = 0.5	38.5	88.9	OR = 0.08, 95% CI = 0.01–0.4, *P = 0.0005

*F* female, *M* Male, *Ma* *Metastrongylus apri*, *Ms* *Metastrongylus salmi*, *Mp* *Metastrongylus pudendotectus*, *n* number of animals, *PR* Paraná, *Pr* prevalence, *RS* Rio Grande do Sul, *SP* São Paulo

*Statistically significant

**Table 4:** Mann–Whitney *U* test results for comparison of mean intensities between sex of hosts and age group of wild boars hunted in São Paulo, Paraná, and Rio Grande do Sul states, Brazil

	Sex			Age group		Statistical analysis
	Male (*n* = 33)	Female (*n* = 25)	Statistical analysis	Juvenile (*n* = 13)	Adult (*n* = 45)	
	Mean intensity (range of intensity)	Mean intensity (range of intensity)		Mean intensity (range of intensity)	Mean intensity (range of intensity)	
Ms	17.5 (1–53)	12.5 (1–58)	*U*_(18)_ = 161, *Z* = 1.4, *P* = 0.1	15 (1–49)	15.3 (1–58)	*U*_(18)_ = 75.5, *Z* = 0.4, *P* = 0.6
Ma	12 (4–26)	16 (3–29)	*U*_(18)_ = 8, *Z* = 1.7, *P* = 0.09	0	12.8 (3–29)	NA
Mp	18.1 (1–59)	32 (1–93)	*U*_(18)_ = 10.5, *Z* = 1.5, *P* = 0.1	0	21.6 (1–93)	NA
Total	24.6 (1–96)	19.6 (1–128)	*U*_(18)_ = 189.5, *Z* = 1.2, *P* = 0.2	15 (1–49)	23.7 (1–128)	*U*_(18)_ = 76.5, *Z* = 0.8, *P* = 0.4

*Ma* *Metastrongylus apri*, *Ms* *Metastrongylus salmi*; Mp *Metastrongylus pudendotectus*, *NA* not applicable, *n* number of animals

### Morphological descriptions

**Fig. 2:**
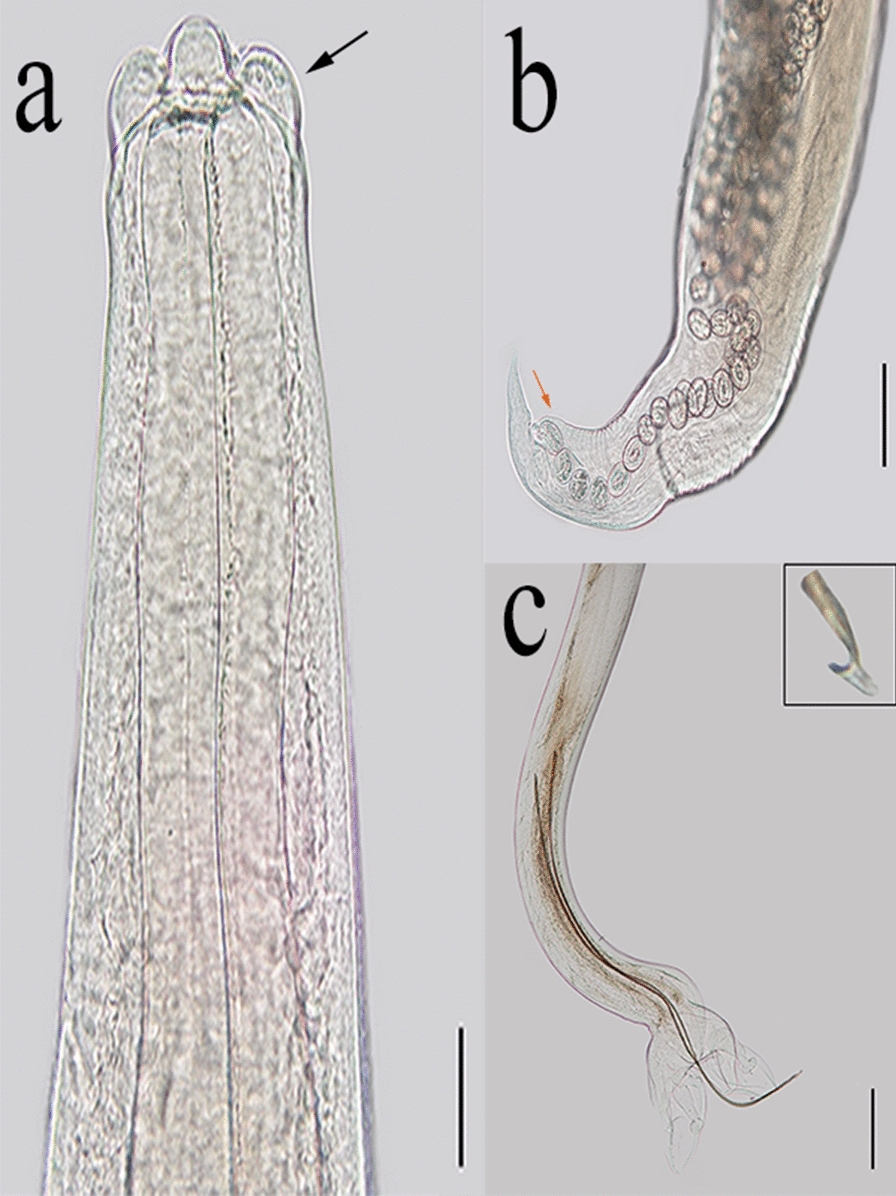
*Metastrongylus salmi* found in wild boars hunted in São Paulo, Paraná, and Rio Grande do Sul states. **a** Anterior extremity, showing the trilobated lips (black arrow). Magnification: ×200. Bar: 50 µm. **b** Female posterior extremity; note the short pre-vulvar swelling (orange arrow). Magnification: ×100. Bar: 100 µm. **c** Male posterior extremity showing the hook-like form at spicule ending (inset). Magnification: ×40. Bar: 2000 µm

*Metastrongylus salmi* Gedoelst [29]—Fig. 2a, b, c.

General description: Thin, long, whitish nematodes in vivo. Anterior extremity composed of trilobed lips and claviform esophagus. Males with short copulatory bursa, with some digitiform rays. Intermediate spicules, similar in size, with transverse striations and a single hook-like form at the end. Gubernaculum is absent. Females with the posterior extremity curved ventrally, conical tail, and vulvar opening close to anus covered by a short swelling. Irregular, double-shelled, and embryonated eggs.

*Habitat *bronchi and bronchioles.

*Host* *Sus scrofa.*

Morphometric data (in millimeters).

Males (*n* = 149).

Total length: 18.7 ± 2.0 (8.9–22.8); width (esophagus–intestinal junction): 0.1 ± 0,0. (0.1–0.2); esophagus: 0.5 ± 0.05 (0.4–0.7); excretory pore (distance for anterior extremity): 0.4 ± 0.04 (0.2–0.5); nerve ring (distance for anterior extremity): 0.3 ± 0.05 (0.2–0.5); large spicule: 2.05 ± 0.1 (1.5–2.6); small spicule: 1.9 ± 0.1 (1.3–2.2).

Females (*n* = 158).

Total length: 42.2 ± 5.2 (27.8–51.3); width (esophagus–intestinal junction): 0.2 ± 0.02 (0.2–0.4); esophagus: 0.7 ± 0.1 (0.5–0.8); excretory pore (distance for anterior extremity): 0.4 ± 0.1 (0.2–0.6); nerve ring (distance for anterior extremity): 0.4 ± 0.1 (0.2–0.6); vulva–posterior extremity: 0.1 ± 0.02 (0.1–0.2); anus–posterior extremity: 0.1 ± 0.01 (0.1–0.2); eggs (length in micrometers): 49.7 ± 2.9 (43.0–64.0); eggs (width in micrometers): 35.3 ± 2.7 (30.0–40.5).

**Fig. 3:**
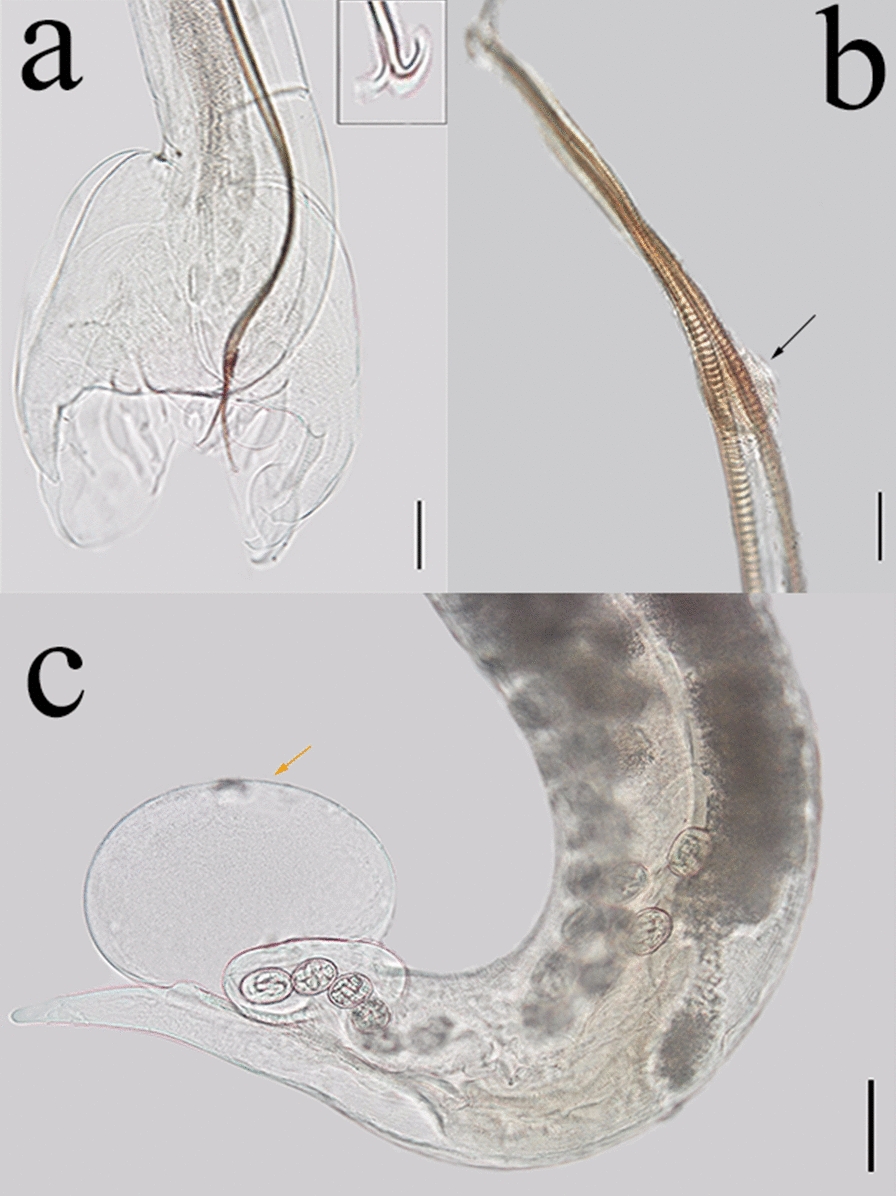
*Metastrongylus pudendotectus* found in wild boars hunted in Paraná and Rio Grande do Sul states. **a** Male posterior extremity; note the anchor-like form at the spicule ending (inset). Magnification: ×100. Bar: 100 μm. **b** Spicule and gubernaculum (black arrow). Magnification: x400. Bar: 25 μm. **c** Female posterior extremity; note the prominent swelling surrounding the vulvar opening (orange arrow). Magnification: x100. Bar: 100μm

*Metastrongylus pudendotectus* Vostokov [30]—Fig. 3(a, b, c).

General description: Thin, long, whitish nematodes in vivo. Mouth with trilobed lips and claviform esophagus. Males with large copulatory bursa and short rays. Short spicules, when compared to other species of the genus, with transverse striations and anchor-like form at the end. Gubernaculum is present. Female with posterior extremity curved ventrally, conical tail, vulvar opening close to the anus covered by a prominent swelling. Irregular, double-shelled, and embryonated eggs.

*Habitat* bronchi and bronchioles.

*Host* *Sus scrofa.*

Morphometric data (in millimeters).

Males (*n* = 26).

Total length: 19.2 ± 1.7 (16.2–23.5); width (esophagus–intestinal junction): 0.1 ± 0,01 (0.1–0.2); esophagus: 0.5 ± 0.03 (0.5–0.6); excretory pore (distance for anterior extremity): 0.3 ± 0.03 (0.2–0.4); nerve ring (distance for anterior extremity): 0.3 ± 0.03 (0.2–0.4); large spicule: 1.5 ± 0.1 (1.3–1.7); small spicule: 1.4 ± 0.05 (1.3–1.5); gubernaculum (length): 0.04 ± 0.007 (0.03–0.1); gubernaculum (width): 0.02 ± 0.008 (0.01–0.04).

Females (*n* = 30).

Total length: 33.0 ± 5.5 (20.4–52.0); width (esophagus–intestinal junction): 0.2 ± 0.03 (0.1–0.3); esophagus: 0.6 ± 0.1 (0.4–0.8); excretory pore (distance for anterior extremity): 0.3 ± 0.05 (0.2–0.5); nerve ring (distance for anterior extremity): 0.3 ± 0.05 (0.2–0.4); vulva–posterior extremity: 0.2 ± 0.04 (0.1–0.3); anus–posterior extremity: 0.1 ± 0.02 (0.1–0.2); eggs (length in micrometers): 60.7 ± 5.6 (44.3–76.3); eggs (width in micrometers): 44.6 ± 2.3 (37.0–52.0).

**Fig. 4:**
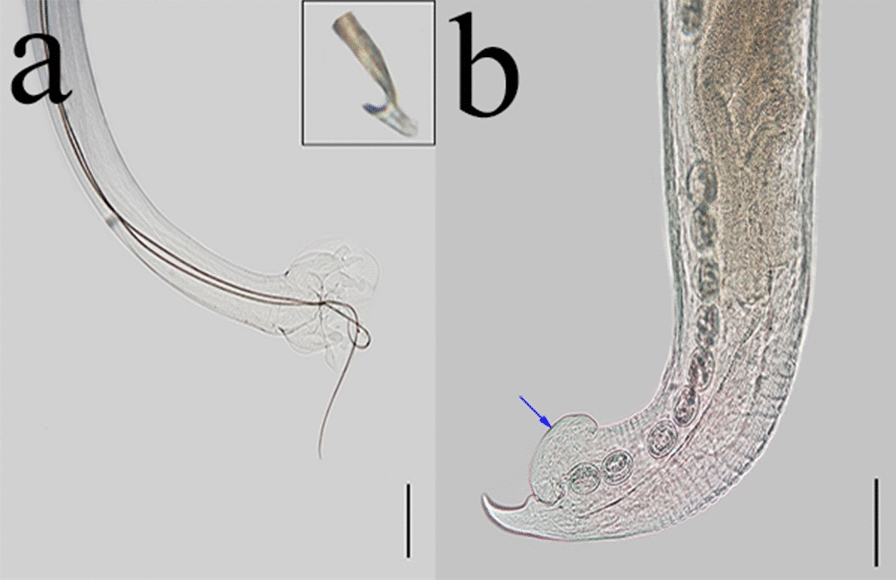
*Metastrongylus apri* found in wild boars hunted in Paraná and Rio Grande do Sul states. **a** Male posterior extremity showing the hook-like form at the spicule ending (inset). Magnification: ×40. Bar: 2000 µm. **b** Female posterior extremity; note the protruding vulvar swelling (blue arrow). Magnification: ×100. Bar: 100 µm

*Metastrongylus apri* Gmelin [31]—Fig. 4 (a, b).

General description: Thin, long, whitish nematodes in vivo. Mouth with two trilobed lips and claviform esophagus. Male with copulatory bursa with broad edges and a rounded lateral external ray in a mushroom-like form. Very long spicules, similar in length, with a single hook-like ending. Gubernaculum is absent. Female with posterior extremity curved ventrally, conical tail, vulvar opening close to the anus covered by a vulvar swelling with an intermediate length between *M. salmi* and *M. pudendotectus*. Irregular, double-shelled, and embryonated eggs.

*Habitat* bronchi and bronchioles.

*Host* *Sus scrofa.*

Morphometric data (in millimeters).

Males (*n* = 33).

Total length: 18.4 ± 1.8 (14.6–21.4); width (esophagus–intestinal junction): 0.1 ± 0.01 (0.1–0.2); esophagus: 0.5 ± 0.1 (0.4–0.6); excretory pore (distance for anterior extremity): 0.4 ± 0.04 (0.3–0.4); nerve ring (distance for the anterior extremity): 0.3 ± 0.04 (0.2–0.4); large spicule 4.5 ± 0.2 (4.2–5.3); small spicule: 4.3 ± 0.2 (3.9–4.7).

Females (*n* = 33).

Total length: 43.4 ± 6.1 (26.4–51.0); width (esophagus–intestinal junction): 0.2 ± 0.01 (0.2–0.3); esophagus: 0.70 ± 0.1 (0.6–0.9); excretory pore (distance for anterior extremity): 0.5 ± 0.1 (0.3–0.6); nerve ring (distance for anterior extremity): 0.4 ± 0.1 (0.3–0.6); vulva–posterior extremity: 0.1 ± 0.02 (0.1–0.2); anus–posterior extremity: 0.1 ± 0.01 (0.1–0.2); eggs (length in micrometers): 53.3 ± 3.0 (44.9–58.4); eggs (width in micrometers): 39.9 ± 2.1 (34.2–46.3).

### PCR and phylogenetic analysis

**Fig. 5:**
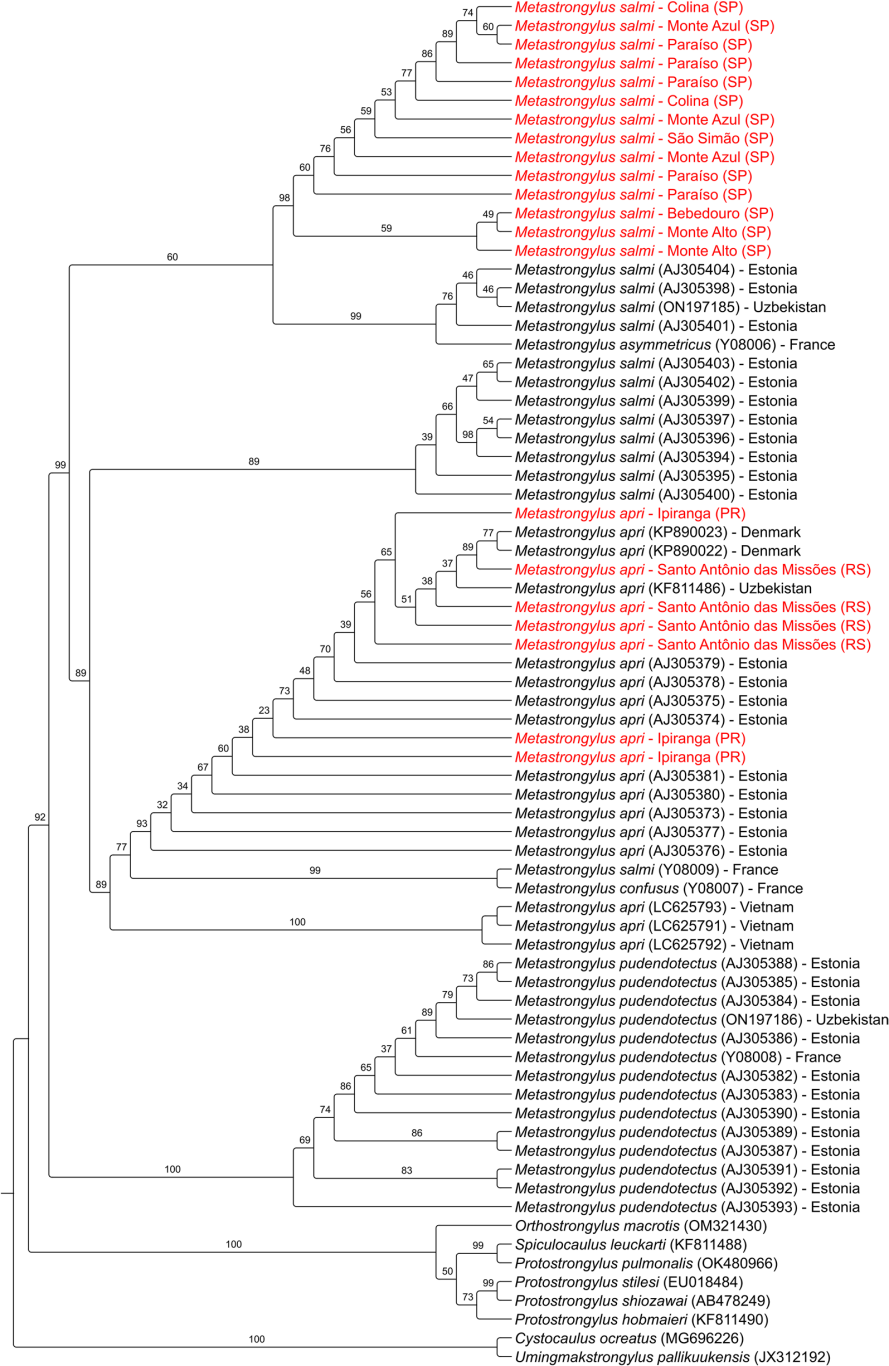
Maximum-likelihood tree using internal transcribed spacer (ITS) region encompassing Metastrongyloidea helminths. *Cystocaulus ocreatus* and *Umingmakstrongylus pallikuukensis* were rooted as outgroups. Sequences obtained from the study are highlighted in red. *Metastrongylus* sequences downloaded from the GenBank are indicated with accession number, species name, and country. Bootstrap values are shown at the nodes. The best-fit model was the transversion model with equal base frequencies and the discrete Gamma model with four rate categories (TVMe + G4)

**Fig. 6:**
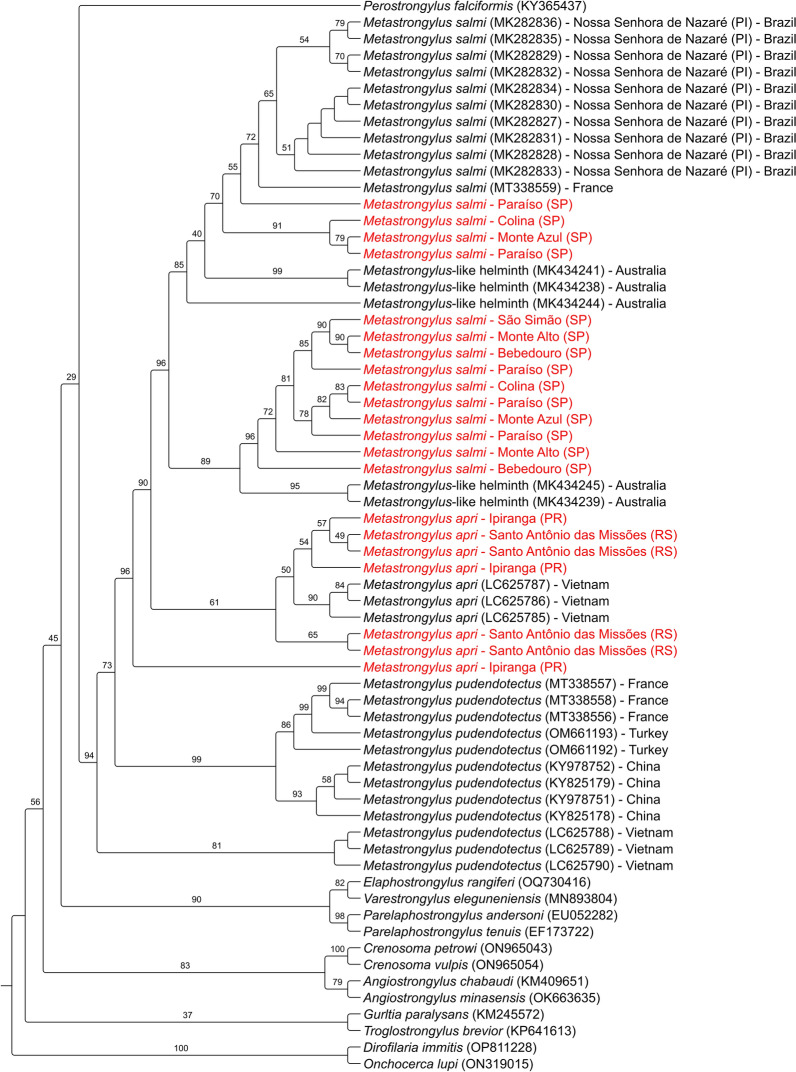
Maximum-likelihood tree using cytochrome *c* oxidase subunit 1 mitochondrial DNA (*cox-1* mtDNA) region encompassing Metastrongyloidea helminths. *Dirofilaria immitis* and *Onchocerca lupi* were rooted as outgroups. Sequences obtained from the study are highlighted in red. Nucleotide sequences downloaded from the GenBank are indicated with accession number, species name, and country. Bootstrap values are shown at the nodes. The best-fit model was the transition model considering the base frequencies, invariable sites, and discrete Gamma model with four rate categories (TIM + F + I + G4)

*Metastrongylus salmi* and *M. apri* genetic material were amplified by at least two out of the four genetic markers used. *Metastrongylus pudendotectus* samples yielded low DNA and could not be used for phylogenetic analysis. The amplicons varied from 784 to 924 base pairs (bp) for 18S rDNA; 623 to 687 for 28S rDNA; 231 to 509 bp for the ITS region, and 626 to 702 bp for *cox-1* mtDNA. BLAST analysis revealed disagreements between morphological identification and genetic data (Table 5). The phylogenetic trees for the 18S rDNA, 28S rDNA, ITS region, and *cox-1* mtDNA can be seen in Additional file 1, Additional file 2 and Fig. 5, 6.

**Table 5:** Basic Local Alignment Search tool (BLAST) results compared with morphological identification of *Metastrongylus* species found in wild boars hunted in São Paulo, Paraná, and Rio Grande do Sul states, Brazil

Sample	Location	^a^Percentage identity/query cover				Morphological identification
		18S	28S	ITS	*cox-1*	
31	São Simã (SP)	99.9% Ma/100 99.9% Ms/100%	98.3% Ma/100% 98.5% Ms/98%	95.1% Ms/100%	96.1% Ms/99%	Ms
46	Colina (SP)	100% Ma/100%100% Ms/100%	98.4% Ma/99% 98.5% Ms/96%	94.4% Ms/100%	96.0% Ms/100%	Ms
47	Colina (SP)	100% Ma/100% 100% Ms/ 100%	98.2% Ma/100% 98.3% Ms/98%	95.3% Ms/100%	99.8% Ms/100%	Ms
50	Monte Azul (SP)	100% Ma/100% 100% Ms/100%	98.4% Ma/99%98.5% Ms/ 96%	94.4% Ms/100%	96.1% Ms/99%	Ms
51	Monte Azul (SP)	100% Ma/100% 100% Ms/100%	NA	93.9% Ms/100%	99.8% Ms/99%	Ms
52	Monte Azul (SP)	100% Ma/100% 100% Ms/100%	98.2% Ma/100% 98.5% Ms/ 96%	94.9% Ms/100%	96.1% Ms/100%	Ms
61	Paraíso (SP)	100% Ma/100% 100% Ms/100%	98.1% Ma/99% 98.2% Ms/ 96%	94.1% Ms/100%	99.8% Ms/99%	Ms
62	Paraíso (SP)	100% Ma/100% 100% Ms/100%	98.4% Ma/99% 98.63% Ms/96%	95.0% Ms/100%	96.1% Ms/99%	Ms
63	Paraíso (SP)	99.9% Ma/100% 99.9% Ms/100%	98.5% Ma/100% 98.6% Ms/ 98%	95.1% Ms/100%	96.1% Ms/99%	Ms
68	Paraíso (SP)	100% Ma/100% 100% Ms/100%	98.5% Ms/100% 98.3% Ma/ 100%	95.3% Ms/99%	96.1% Ms/99%	Ms
69	Paraíso (SP)	100% Ma/100% 100% Ms/100%	98.3% Ma/99% 98.5% Ms/98%	95.4% Ms/99%	99.8% Ms/100%	Ms
76.1	Ipiranga (PR)	100% Ma/100% 100% Ms/100%	99.7% Ma/100%	100% Ma/100%	90.6% Ms/100%	Ma
76.2	Ipiranga (PR)	100% Ma/100% 100% Ms/100%	99.6% Ma/99% 98.9% Ms/ 96%	100% Ma/98%	94.4% Ms/100%	Ma
76.3	Ipiranga (PR)	100% Ma/100% 100% Ms/100%	99.7% Ma/100% 99.0% Ms/100%	100% Ma/98%	94.5% Ms/99%	Ma
79	Bebedouro (SP)	100% Ma/100% 100% Ms/100%	98.2% Ms/100% 98.0% Ma/100%	93.9% Ms/100%	95.9% Ms/99%	Ms
80.1	Santo Antônio das Missões (RS)	NA	99.6% Ma/100% 98.9% Ms/98%	100% Ma/100%	94.6% Ms/99%	Ma
80.2	Santo Antônio das Missões (RS)	NA	100% Ma/100% 99.4% Ms/97%	100% Ma/100%	94.4% Ms/99%	Ma
80.3	Santo Antônio das Missões (RS)	NA	99.5% Ma/100% 98.9% Ms/100%	100% Ma/ 100%	94.5% Ms/ 99%	Ma
80.4	Santo Antônio das Missões (RS)	NA	NA	99.7% Ma/98%	94.9% Ms/99%	Ma
95.1	Monte Alto (SP)	100% Ms/98% 100% Ma/ 96%	98.4% Ms/99% 98.3% Ma/99%	96.9% Ms/ 95%	96.0% Ms/100%	Ms
95.2	Monte Alto (SP)	NA	98.2% Ma/100%98.3% Ms/99%	93.7% Ms/100%	96.1% Ms/100%	Ms

^a^The percentage identity and query cover refers to the first two BLAST results for 18S and 28S and the first for ITS and *cox-1*. *Ma* *Metastrongylus apri*, *Ms* *Metastrongylus salmi*, *NA* not amplified

## Discussion

The prevalence of *Metastrongylus* spp. observed in the present study was higher when compared with studies with wild boars from commercial breeding [32, 33] and free-ranging animals [34] from Brazil. However, the percentage is lower compared to European countries, where prevalence values are greater than 80% [35]. The different results could be related to animal density, different climatic and terrains of each region, and earthworm distribution. Areas with grain and sugarcane plantations provide abundant food to wild boars, representing an increased risk of infection due to increased final host density and, therefore, a larger population of infected earthworms [36]. In addition, in São Paulo state, food bait is provided in some hunting areas (EGL Hoppe, personal communication, June 24, 2023) and could be related to the high prevalence (77.5%) observed in the region, despite the obvious differences between sample sizes and climate. The construction of fences can also lead to a concentration of animals in a certain area and thus increases the number of infective stages in the environment [37]. A higher prevalence of *Metastrongylus* spp. was found in areas with high altitude and abundant rainfall, suggesting that such conditions may improve the resistance of parasite eggs and the survival of the intermediate host [38]. The distribution of earthworms is similar to the hosts’ geographical range, but in hot and dry climates, their number could be reduced [39]. The viability of the embryonated egg can be influenced by several climatic factors, and the dryness should be the most important. Moist soil environments can lead to an increased lifespan of the eggs, surviving for 2 years or more [40].

Infection with *Metastrongylus* spp. (OR = 0.1, 95% CI = 0.02–0.6, *P* = 0.004) and *M. salmi* (OR = 0.08, 95% CI = 0.01–0.4, *P* = 0.0005) was significantly higher in adults than in juveniles. However, some evidence demonstrates a higher prevalence of nematodes and digenetic trematodes in younger animals [35, 38, 41, 42], whereas others show no correlation between age and prevalence of lungworms [7, 36, 43]. The higher prevalence observed in the present study may be related to an accumulative effect caused by a greater or more prolonged chance of exposure in older animals over time [44], a well-documented factor in parasites of wild rodents from Europe [45].

Differences in the community of *Metastrongylus* spp. can change considerably between countries and, within the same country, between regions [5]. A study with wild boars in commercial breeding in southern Brazil observed a predominance of *M. apri* (52.5%), followed by *M. salmi* (20%), and *M. pudendotectus* (7.5%) [33]. In two properties of São Paulo state, wild boars were infected with *M. salmi* (50% and 15.2%) and *M. pudendotectus* (5.6% and 3%) [32], and in another survey in wild boars from the municipality of Monte Azul, one of the sampling sites present in this study, *M. salmi* (82.9%) and *M. pudendotectus* (11.4%) [34] were recovered. This variation may be restricted to a local level since, in the present study, only *M. salmi* was found in the São Paulo municipalities, and in other studies, two different species were reported [32, 33]. The absence of *M. pudendotectus* in the municipalities of São Paulo may also be associated with its low prevalence in the state, making it difficult to detect in small sample sizes.

The different distribution of species observed in São Paulo state (only *M. salmi*) and the states of southern Brazil (*M. salmi*, *M. apri*, and *M. pudendotectus*) may be associated with the different climatic conditions between these regions or with the low sample size in the studied sites. Wild boars kept in a subtropical climate, present throughout the southern region, may be infected by different species of helminths and demonstrate other infection parameters when compared to animals that live in other climatic conditions [33]. It is important to note that the increase in the host population contributes to the structure of the parasite community in a given region, but its composition can change with the introduction of new parasites in native populations. Such an event may be a consequence, for example, of the translocation of wild boars for hunting purposes [46, 47], one of the practices responsible for expanding the wild boar population in Brazil [48].

Wild boars in southern (Paraná and Rio Grande do Sul) and southeastern (where São Paulo is located) regions may have different origins, and this would be reflected in the different *Metastrongylus* communities observed in the present study. The second wave of the invasion of wild boars in Brazil came from Uruguay to the south of Rio Grande do Sul [49]. In the 1990s, several commercial breedings were established in the southern and southeastern regions with animals imported from Europe and Canada [3]. Unintentional or deliberated release of half-bred or pure wild boars in a rearing farm located in the municipality of Piedade, São Paulo state, might have contributed to their expansion in the region [3, 50]. Interestingly, one study found *M. apri* and *M. pudendotectus* in free-ranging wild boars from the northern region of Uruguay [FN Inzaguirre Pomponi and CS Nuñez de Moraes Gomez, PhD dissertation],^1^ the same two species reported in the southern states.

Despite being the second most important helminth disease in domestic swine breeding, second only to *Ascaris suum* infection, metastrongylosis in intensive regimes has reduced significantly over the years because the animals are kept on cement floors, which makes contact with the intermediate host improbable. [33]. However, in outdoor farming of domestic swine and wild boar, the disease must be considered, since the environmental conditions and the abundance of hosts favors the maintenance of the nematodes cycle and may cause death or reduced fitness of affected animals, especially when associated with infectious–parasitic agents [51, 52]. The present study describes the presence of infection in free-ranging wild boars, which reinforces the need for effective control measures to avoid the introduction of the helminth in such rearing models.

The phylogenetic analysis revealed that 18S rDNA and 28S rDNA cannot discriminate *Metastrongylus* spp. species, with ITS region and *cox-1* mtDNA proved to be the most suitable markers. A study with domestic pigs in Vietnam was able to separate *M. salmi*, *M. apri*, and *M. pudendotectus* using *cox-1* and ITS2 locus [53]. High-resolution restriction fragment length polymorphism (RFLP) and mutation scanning microsatellite assays with ITS2 region found intraspecific and interspecific variation between *Metastrongylus* isolates, supporting the utility of this marker for nematode species identification [54, 55]. Mitochondrial genes such as *cox-1* have a faster evolutionary rate and, for this reason, should be suitable for discriminating closely related species when compared with nuclear ribosomal genes [56]. The partial gene *cox-1* was able to properly distinguish *M. salmi* eggs recovered from pigs of Piaui state, northeastern Brazil [8].

Interestingly, the *cox-1* tree shows that *M. salmi* used in the study can present considerable genetic diversity, with some isolates forming a highly supported clade with species from Australia, France, and Brazil. The ITS2 and *cox-1* trees also revealed some close relations between *M. apri* and others from Europe and Asia but with lower bootstrap values. In the same country of the study, three haplotypes from *M. salmi* were reported: two new and one for European countries [8]. Further studies should be performed to better understand *Metastrongylus* genetic diversity in Brazil.

We could not explain the differences between morphological and genetic data observed in Table 5. This may be related to the scarcity of genetic markers available in the GenBank database or the close genetic relationship between *M. apri* and *M. salmi* (interspecific nucleotide variation between 1.3 and 3.6%) [53]. Morphological misidentification should be ruled out because *M. salmi* can be easily distinguished from *M. apri* by the length of spicules (shorter than *M. apri*) and less pronounced pre-vulvar swelling in females [6].

Our results highlight the importance of studying helminths from different localities to explore better the environmental diversity and their influence on the nematode population. Further studies to determine *Metastrongylus* ecology in other Brazilian biomes would help to assess the relationship between the lungworm communities and the host population in the country.

## Conclusions

The present study shows differences in *Metastrongylus* communities from São Paulo and the southern states and shows for the first time that wild boars can act as a source of *Metastrongylus* infection for domestic and wild animals. Given the scarcity of the nematode genetic data in databases, we expanded the sequences available for *M. salmi* in addition to other genetic markers explored, and presented novel sequences from *M. apri*. These new genetic data will help further studies to understand the genetic variability of *Metastrongylus* nematodes in different regions.

### Supplementary Information

**Additional file 1: ** Maximum-likelihood tree using 18S ribosomal DNA region encompassing Metastrongyloidea superfamily helminths. *Syngamus trachea* and *Necator americanus* were rooted as outgroups. Sequences obtained from the study are highlighted in red. *Metastrongylus* sequences downloaded from the Genbank are indicated with accession number, species name, and country. Bootstrap values are shown at the nodes. The best-fit model was Tamura two parameters considering the base frequencies and invariable sites (TPM2+F+I).

**Additional file 2: ** Maximum-likelihood tree using 28S ribosomal DNA region encompassing Metastrongyloidea superfamily helminths. *Toxocara vitulorum* and *Toxocara canis* were rooted as outgroups. Sequences obtained from the study are highlighted in red. *Metastrongylus* sequences downloaded from the Genbank are indicated with accession number, species name, and country. Bootstrap values are shown at the nodes. The best-fit model was the transversion model with equal base frequencies and the discrete Gamma model with four rate categories (TVMe+G4).

## Data Availability

All data generated during this study are included in the published article.

## Abbreviations

PBS: Phosphate-buffered saline
ATL: Tissue lysis buffer
DNA: Deoxyribonucleic acid
18S rDNA: 18 Subunit of ribosomal deoxyribonucleic acid
28S rDNA: 28 Subunit of ribosomal deoxyribonucleic acid
ITS: Internal transcribed spacer
*cox-1* mtDNA: Cytochrome *c* oxidase subunit 1 of mitochondrial deoxyribonucleic acid
Taq DNA: *Thermus aquaticus* deoxyribonucleic acid
PCR: Polymerase chain reaction
BLAST: Basic Local Alignment Search Tool
bp: Base pairs

## Footnotes

1 Izaguirre Pomponi FN, Nuñez de Moraez Gomes CS. Estudio de la helmintofauna de jabalíes y cerdos asilvestrados (*Sus scrofa*) de la región norte de Uruguay [doctoral dissertation]. Universidad de la República; 2022.
