# Supplementary figures and images for: Integrative taxonomy of Metastrongylus spp. in wild boars from Brazil
Source: Parasit Vectors. 2023 Dec 5;16:449. doi: 10.1186/s13071-023-06047-x (PMC10696852; doi:10.1186/s13071-023-06047-x)

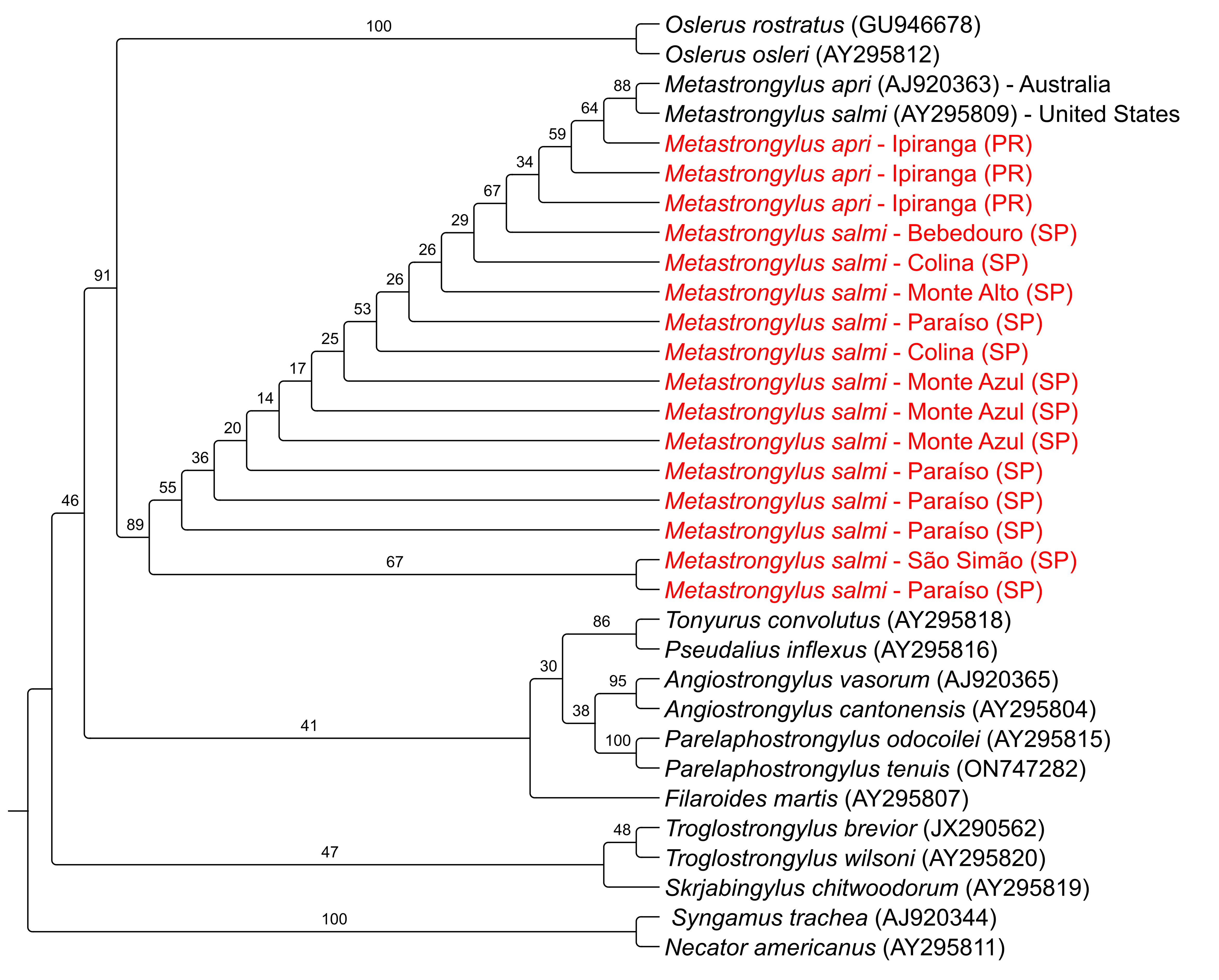

Supplement: Supplementary file 1 — Additional file 1: Maximum-likelihood tree using 18S ribosomal DNA region encompassing Metastrongyloidea superfamily helminths. Syngamus trachea and Necator americanus were rooted as outgroups. Sequences obtained from the study are highlighted in red. Metastrongylus sequences downloaded from the Genbank are indicated with accession number, species name, and country. Bootstrap values are shown at the nodes. The best-fit model was Tamura two parameters considering the base frequencies and invariable sites (TPM2+F+I). [file 13071_2023_6047_MOESM1_ESM.tif]

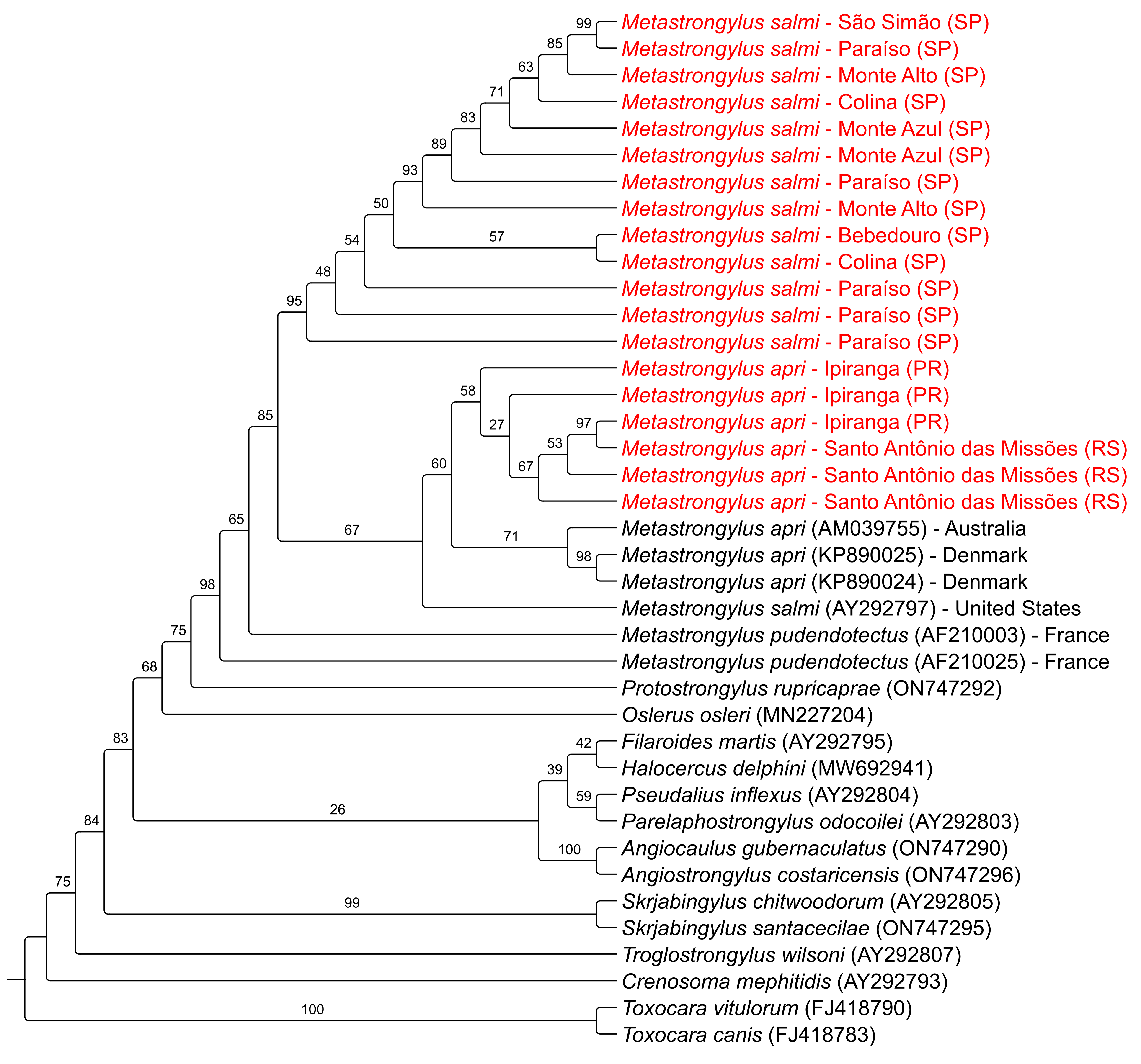

Supplement: Supplementary file 2 — Additional file 2: Maximum-likelihood tree using 28S ribosomal DNA region encompassing Metastrongyloidea superfamily helminths. Toxocara vitulorum and Toxocara canis were rooted as outgroups. Sequences obtained from the study are highlighted in red. Metastrongylus sequences downloaded from the Genbank are indicated with accession number, species name, and country. Bootstrap values are shown at the nodes. The best-fit model was the transversion model with equal base frequencies and the discrete Gamma model with four rate categories (TVMe+G4). [file 13071_2023_6047_MOESM2_ESM.tif]
